# Study of Water-Based Lithium Titanate Electrode Processing: The Role of pH and Binder Molecular Structure

**DOI:** 10.3390/polym8080276

**Published:** 2016-08-02

**Authors:** Diogo Vieira Carvalho, Nicholas Loeffler, Guk-Tae Kim, Mario Marinaro, Margret Wohlfahrt-Mehrens, Stefano Passerini

**Affiliations:** 1 Helmholtz Institute Ulm (HIU), Helmholtzstrasse 11, 89081 Ulm, Germany; diogo.carvalho@kit.edu (D.V.C.); nicholas.loeffler@kit.edu (N.L.); 2 Karlsruhe Institute of Technology (KIT), P.O. Box 3640, 76021 Karlsruhe, Germany; 3 Zentrum für Sonnenenergie- und Wasserstoff-Forschung Baden-Württemberg (ZSW), Helmholtzstrasse 8, 89081 Ulm, Germany; mario.marinaro@zsw-bw.de (M.M.); margret.wohlfahrt-mehrens@zsw-bw.de (M.W.-M.)

**Keywords:** lithium batteries, lithium titanate LTO, water-soluble binders, CMC, guar gum, pectin

## Abstract

This work elucidates the manufacturing of lithium titanate (Li_4_Ti_5_O_12_, LTO) electrodes via the aqueous process using sodium carboxymethylcellulose (CMC), guar gum (GG) or pectin as binders. To avoid aluminum current collector dissolution due to the rising slurries’ pH, phosphoric acid (PA) is used as a pH-modifier. The electrodes are characterized in terms of morphology, adhesion strength and electrochemical performance. In the absence of phosphoric acid, hydrogen evolution occurs upon coating the slurry onto the aluminum substrate, resulting in the formation of cavities in the coated electrode, as well as poor cohesion on the current collector itself. Consequently, the electrochemical performance of the coated electrodes is also improved by the addition of PA in the slurries. At a 5C rate, CMC/PA-based electrodes delivered 144 mAh·g^−1^, while PA-free electrodes reached only 124 mAh·g^−1^. When GG and pectin are used as binders, the adhesion of the coated layers to the current collector is reduced; however, the electrodes show comparable, if not slightly better, electrochemical performance than those based on CMC. Full lithium-ion cells, utilizing CMC/PA-made Li[Ni_0.33_Mn_0.33_Co_0.33_]O_2_ (NMC) cathodes and LTO anodes offer a stable discharge capacity of ~120 mAh·g^−1^_(NMC)_ with high coulombic efficiencies.

## 1. Introduction

The worldwide trend to develop light weight and high performance devices, such as smartphones, personal computers and tablets, demands designable and reliable power supplies. In this respect, lithium-ion batteries (LIBs) are well-established systems to power such devices. Additionally, LIBs are also used as the main or secondary energy supply, respectively, in pure electric or hybrid vehicles, thus reducing the dependence of fossil fuels.

Nowadays, a major requirement of new battery technologies is, besides key factors, like high performance, safety and low cost, the sustainability and environmental friendliness of the used components [1,2]. From this point of view, aqueous processing of LIB electrodes, i.e., utilizing only water-soluble binders, is an extremely promising approach. The binder is a fundamental electrode component, since it ensures a firm binding of the active material and the conductive carbon, as well as of the electrode layer to the current collector. Moreover, binders have the additional function to provide appropriate slurry rheology for coating. The state of the art binders for LIBs are poly(vinylidene-di-fluoride) (PVdF) for cathodes and styrene-butadiene rubber (SBR)/sodium-carboxymethylcellulose (CMC) mixtures for graphite-based anode electrodes. Despite being well established in commercial electrodes, the synthetic PVdF binder exhibits some drawbacks, for instance the higher cost compared to natural binders and the necessity of using toxic solvents, particularly *N*-methyl-2-pyrrolidon (NMP), during electrode processing. As an alternative, aqueous processed electrodes are under development, offering significantly-reduced electrode costs and an environmentally-friendly process without compromising the battery performance [3].

Aside from graphite, spinel-structured lithium titanate Li_4_Ti_5_O_12_ (LTO) is commercially used as the anode for LIBs, providing remarkable advantages, such as stable cycling performance even at high C-rates, low toxicity and enhanced safety [4]. Those characteristics render LTO suitable for large-scale LIBs as those used in electric and hybrid vehicles. Moreover, the combination of the LTO desirable properties with the aqueous electrode manufacturing would lead to LIB anodes with enhanced safety and performance properties. Nevertheless, the alkaline nature of aqueous LTO slurries favors the corrosion of the aluminum current collector upon electrode coating [5]. To avoid this issue, the use of mild acids, i.e., phosphoric (PA) and formic (FA) acids, as pH modifiers of Li[Ni_0.33_Mn_0.33_Co_0.33_]O_2_ (NMC) electrodes has been earlier investigated in our laboratories [6]. It was reported that both acids can reduce the pH of the electrode slurry to values of about nine, thereby preventing corrosion. Moreover, better cyclability was shown for electrodes prepared with the addition of PA due to the formation and deposition of highly insoluble phosphate compounds on the active material surface of NMC, which reduce the transition metal leaching and enhance the electrochemical performance.

As an alternative to SBR/CMC and PVdF binders, natural polysaccharides, such as guar gum (GG), have been investigated as potential binders for graphite [7], silicon [8,9] and LTO [10] electrodes. Yoon et al. reported the use of pectin as a binder for silicon anodes and compared their electrochemical performance with CMC and amylose-based electrodes correlating the electrochemical performance, in particular cycle life, with the polysaccharides’ backbone structure [11]. In a different approach, this work focuses on the development of an aqueous process to prepare LTO electrodes, using CMC as the binder, but with the addition of phosphoric acid (PA) as the pH modifier. Additionally, we investigated different polysaccharides, i.e., guar gum (GG, branched mannose:galactose 2:1 chain) and pectin (α-linking galacturonic acid chain), as binders for LTO electrodes, assessing the adhesion strength, thermal stability, electrode morphology and electrochemical performance. Finally, we report our investigation of full cells comprising NMC as the cathode and LTO as the anode, both prepared via aqueous processing using natural polymeric binders.

## 2. Materials and Methods

### 2.1. Electrode Processing

Lithium titanate (Li_4_Ti_5_O_12_, Hombitec LTO5; average primary particle size: 250 nm, Huntsman, Duisburg, Germany) and Li[Ni_0.33_Mn_0.33_Co_0.33_]O_2_ (NMC; average particle size d_90_ = 10 µm, TODA, Battle Creek, MI, USA) were used as, respectively, anode and cathode active materials. Sodium carboxymethylcellulose (Walocel CRT 2000 PPA 12, Dow Wolff Cellulosics, Bomlitz, Germany) with a degree of substitution of 1.2, guar gum (GG, Lamberti SpA, Albizzate, Italy) and pectin (from citrus peel, Alfa Aesar, Ward Hill, MA, USA) were used as binders for the anodes. The conducting carbon black was always C-NERGY Super C45 (Imerys, Bironico, Switzerland). NMC electrodes were prepared using only CMC as the binder, but with the addition of PA (Bernd Kraft GmbH, Duisburg, Germany) as the pH modifier [6]. All electrodes (NMC or LTO) were prepared with the same methodology. At first, the binder was dissolved in deionized water by magnetic stirring, and subsequently, a predetermined amount of Super C45 was added. After 3 h of continuous mixing, the active material was added, and the electrode slurries were stirred for 2 h. Further dispersion at medium stirring speed (5000 rpm) was performed with a high-speed mixer (4000-4/65, DREMEL, Mount Prospect, IL, USA). For all electrode formulations (NMC, LTO-CMC-PA, LTO-pectin-PA and LTO-GG-PA), 1% of PA by weight of active material (LTO) was added in the slurry to prevent aluminum current collector corrosion [5,6]. To evaluate the influence of PA, a PA-free, LTO-CMC slurry was also prepared. The slurries were cast on aluminum foil (thickness: 20 µm) using a laboratory-scale doctor blade coater with a wet thickness ranging between 150 and 180 µm. The coated aluminum foils were pre-dried in an atmospheric oven at 80 °C, then under vacuum at 180 °C for 12 h prior to performing electrochemical investigations. The electrode formulation was 88 wt% active electrode material (LTO or NMC), 7 wt% Super C45 and 5 wt% binder. To homogenize the surface, reduce the thickness and, therefore, the porosity, the electrodes were pressed at 10 tons·cm^−2^ using a manual press (Atlas manual hydraulic press 15T, Specac, Orpington, UK) for 30 s. This process also enhanced the adhesion of the active material layer to the current collector.

### 2.2. Electrode Characterization

The thermal properties of CMC, GG, pectin and LTO were evaluated by thermogravimetric analysis (TGA). The TGA experiments were carried out on a Q 5000 IR TGA instrument (TA Instruments, New Castle, DE, USA) by heating the respective specimen from 30 °C up to 500 °C with a heating rate of 5 °C·min^−1^ under a nitrogen gas flow (25 mL·min^−1^) using open aluminum pans. The samples (10–20 mg) were evaluated without any pre-treatment. The electrode morphology was investigated using the ZEISS LEO 1550VP Field Emission SEM (Carl Zeiss, Oberkochen, Germany), while energy dispersive X-ray spectroscopy (EDX) experiments were performed using an EDX X-MaxN (50 mm^2^), 10 kV (Oxford Instruments, Abingdon Oxfordshire, England).

**Figure 1:**
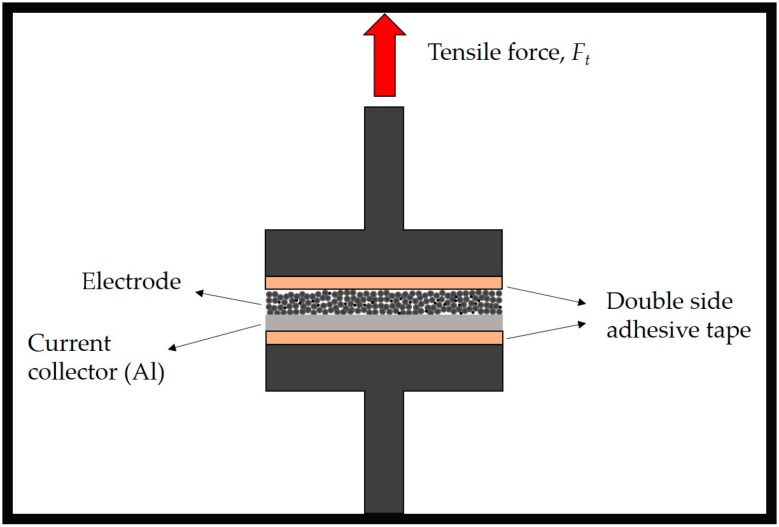
Representation of the adhesion strength measurement.

The electrode adhesion strength was evaluated using a Z2.5 Zwick/Roell machine (Zwick Roell, Ulm, Germany). Figure 1 displays the basic measurement principle. Briefly, an electrode of defined area (6.45 cm^2^) is fixed between two planar and parallel plates with the help of double-sided adhesive tape (3M). After a start phase, in which the specimen is approached before contact is established, the compression phase takes place. Within the compression phase, the compression stress rises until a defined pressure level (2000 N) is achieved and then kept constant during dwell time (120 s), in order to allow the adhesive to contact the electrode. Afterwards, the pull-off phase (1000 mm·min^−1^) takes place, and the maximum tensile force is detected. The adhesion strength $\sigma_{n}$ is calculated, by using Equation (1), from the maximum tensile force |*F*_t, max_| or pull-off force related to the sample area *A*.

(1) $$\sigma_{n}=\frac{\left|F_{t,\text{ max}}\right|}{A}$$

### 2.3. Electrochemical Characterization

Half-cells, i.e., cells made with the Li metal anode (Rockwood Lithium, battery grade, Frankfurt am Main, Germany), were assembled in pouch bag configuration in order to evaluate the effect of PA and the different binders (CMC, GG and pectin) on the LTO electrodes’ performance. The cells were assembled in a dry-room (R.H. <0.01% at 20 °C ± 1 °C), using commercial electrolyte consisting of a 1 mol solution of lithium hexafluorophosphate (LiPF_6_) in a mixture of ethylene carbonate and dimethyl carbonate (EC:DMC (1:1 *w*/*w*)) (LP30, BASF, Ludwigshafen, Germany). The porous polyethylene membrane from Asahi Kasei (Hipore SV718, Tokyo, Japan) was used as the separator. Full-cells were also assembled to evaluate the potential of the aqueous processed electrodes in Li-ion cells. For such tests, coin cells (2032) were assembled in an argon-filled glove box (O_2_ < 0.1 ppm, H_2_O < 0.1 ppm) using NMC and LTO electrode discs (area = 1.13 cm^2^). As the separator, a glass felt (GF/D, Whatman, Maidstone, England) was placed between the electrodes and soaked with the electrolyte (LP30). The cells were tested using a MACCOR Battery tester 4300 (Tulsa, OK, USA) at controlled temperature in climatic chambers (Binder KB 400) at 20 °C ± 0.1 °C. The galvanostatic charge/discharge tests of LTO half-cells were performed between 1.0 V and 2.5 V vs. Li/Li^+^. The galvanostatic tests were performed at different C-rates (0.1C, 0.5C, 1C, 2C, 3C and 5C). The full cell (NMC/LTO) tests were carried out between 1.3 V and 2.8 V.

## 3. Results and Discussion

### 3.1. Thermal Stability

**Figure 2:**
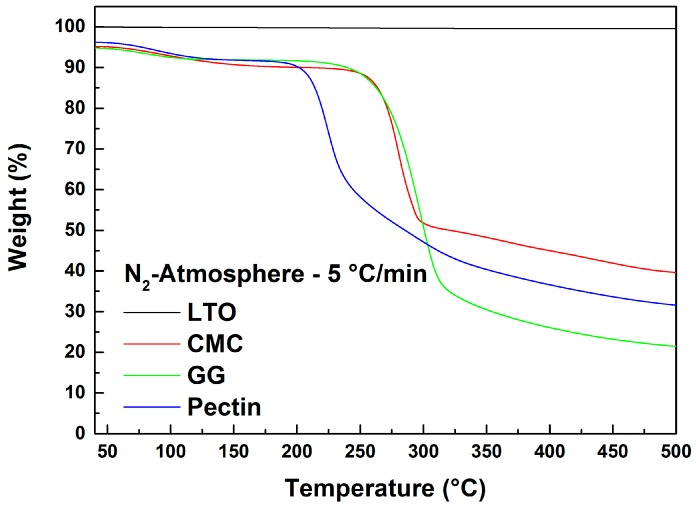
TGA weight loss profiles of LTO, CMC, guar gum (GG) and pectin with a heating rate of 5 °C/min in N_2_ atmosphere.

Figure 2 displays the TGA results in N_2_ atmosphere of LTO, CMC, GG and pectin. LTO particles are stable to 500 °C since no material degradation is detected up to this temperature. The three binders, on the other hand, show comparable weight loss profiles up to 200 °C. The weight decrease at 200 °C is related to water desorption from the polymers since they were not pre-dried. Above 200 °C, pectin exhibits a sharp decomposition, thus showing a lower thermal stability than CMC and GG, in line with previous reports [12]. In contrast, CMC and GG start to decompose only at temperatures above 250 °C [10,13]. The decomposition mechanism of all three polymers is determined by the breakdown of the main polymer chain [7,10,12,13,14]. Overall, all binders showed thermal stability at least up to 200 °C, which allows the high temperature (180 °C) drying of the coated electrode without thermal decomposition. 

### 3.2. Electrode Surface Characterization

**Figure 3:**
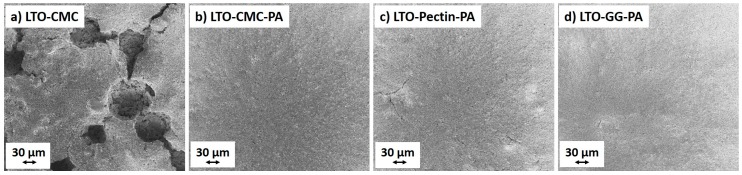
SEM images of unpressed LTO electrodes using: (**a**) CMC as the binder (LTO-CMC); (**b**) CMC as the binder and phosphoric acid (PA) (LTO-CMC-PA); (**c**) pectin as the binder and PA (LTO-pectin-PA); (**d**) and guar gum as the binder and PA (LTO-GG-PA).

SEM images from the unpressed electrodes are shown in Figure 3. Panel (a) shows the micrograph of the LTO-CMC electrode prepared without PA addition. The electrode surface is dominated by cavities generated by the gas evolution, which originates from the reaction of the alkaline slurry with the aluminum current collector during the casting and drying step. In fact, without the addition of PA, the slurry achieves a pH of 11.4 (see Figure S1), leading to aluminum (current collector) corrosion and H_2_ bubble formation [15]. For a detailed investigation of the Al current collector corrosion, energy dispersive X-ray spectroscopy EDX experiments were performed on several spots of the LTO-CMC electrode. Table S1 shows the elemental composition and Figure S2 the SEM micrograph of the spots evaluated. Spots 1 (Spectrum 1) and 2 (Spectrum 2) are in the depth of the cavity, while Spot 3 (Spectrum 3) is on the electrode surface. The high fraction of Al detected on Spots 1 (87.69%) and 2 (66.12%) reveals the exposure of the current collector at the bottom of the observed cavities. In Spot 3, a small fraction of Al was detected (1.2%), which is a side product of the Al corrosion, mainly Al_2_O_3_, which after solubilization from the current collector, is redeposited onto the solid electrode components after solvent evaporation. Thus, the high pH value of the aqueous LTO slurry corrodes the aluminum current collector, and traces of Al can be detected in the composite electrode layer. Its influence will be discussed in the next sections.

On the other hand, the electrodes prepared by adding 1 wt% PA (Figure 3b; LTO-CMC-PA) show no cavities due to the pH adjustment at values around ~6.7, which avoids Al corrosion (see Figure S1). Moreover, Figure 3c,d displays the surface of the electrodes prepared using pectin and GG as binders. Cracks can be observed on the surface of the LTO-pectin-PA electrode. The LTO-GG-PA electrode surface is much more homogeneous than that of LTO-pectin-PA, but small defects can also be detected. The defects on the electrode are associated with the binder shrinkage during the final drying step.

### 3.3. Adhesion Strength

**Figure 4:**
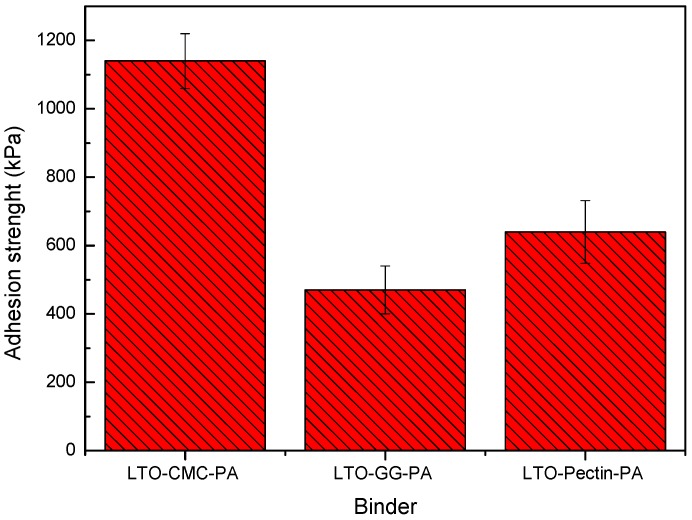
Adhesion strength of LTO electrodes fabricated using PA as an additive and CMC, guar gum and pectin as the binder.

Electrode adhesion strength is a relevant factor for LIBs’ development [16]. The coated composite electrode must, in fact, stand the mechanical stress upon cutting, winding, cell assembling processes and for battery cycle life. Figure 4 displays the adhesion strength of the LTO electrodes. The adhesion strength of the electrode prepared without PA (LTO-CMC) could not be determined, due to cohesion-failure during the pull-off step, i.e., parts of the electrode layer remained on both the adhesive tape and the current collector. This is an extremely negative phenomenon because the delamination of small areas of the electrode layer during processing would reduce the electrode active layer, strongly affecting the cell performance. As depicted in Figure 3a (LTO-CMC), the electrode’s surface morphology is not homogeneous due to the aluminum corrosion. Even after electrode compression (image not shown), the solid particles in the composite electrode do not adhere to each other. On the other hand, the adhesion strength of electrodes coated from PA-containing slurries was successfully measured. The LTO-CMC-PA electrodes exhibited the highest value of adhesion strength (>1100 kPa) compared to the LTO-pectin-PA (>600 kPa) and LTO-GG-PA (>450 kPa) electrodes. The higher adhesion strength of CMC compared to GG electrodes can be explained by the linear β-linkage polymer chain geometry. The interchain hydrogen bonds between the cellulose chains are stronger than between the galactomannan branched chains [10]. Moreover, the linear α-linkage pectin galacturonic acid chain seems to be weaker than the β-linkage of the CMC molecule. In fact, it has been reported that α-linkage polysaccharides are flexible, whereas β-linkage polymers are stiff [11].

### 3.4. Electrochemical Characterization

In order to evaluate the influence of the polymeric binders in LTO electrodes’ performance, half-cells were assembled and tested at several C-rates. In the rate capability test, different current densities are applied to the electrode under investigation, and the corresponding delivered capacities are recorded. Thus, the cells were tested at low (0.1C and 0.5C) and high (2C, 3C and 5C) C-rates (the C-rate is commonly used in battery testing, because it is independent of the electrode active material mass; 1C rate corresponds to a specific current leading to the full discharge/charge of the electrochemical cell in one hour).

**Figure 5:**
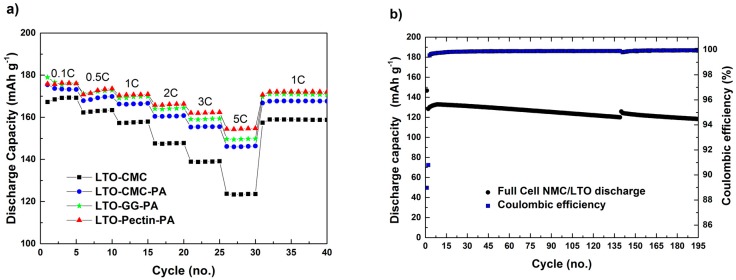
Delivered discharge capacity of (**a**) LTO half-cells using CMC as the binder and CMC, pectin and GG as the binder and PA as an additive at several current densities; LTO mass loading: 3.6–4.1 mg·cm^−2^; electrolyte: 1 mol of LiPF_6_ in ethylene carbonate and dimethyl carbonate (EC:DMC (1:1 *w*/*w*)); and (**b**) cathode-limited Li[Ni_0.33_Mn_0.33_Co_0.33_]O_2_ (NMC)/LTO full-cell using CMC as the binder and PA as an additive at 1C; NMC mass loading: ~4.3 mg·cm^−2^; electrolyte: 1 mol of LiPF_6_ in EC:DMC (1:1 *w*/*w*).

In Figure 5a are depicted the discharge capacities of half-cells assembled with lithium metal and LTO electrodes, these latter incorporating different binders (CMC, CMC-PA, GG-PA and pectin-PA). During the first five cycles at low C-rate (0.1C), the electrodes made from PA-containing slurries showed similar capacity values, while slightly lower discharge capacities were observed for the electrode made without PA. Once the current density increases to 0.5C and subsequently to 1C, the difference in cycling performance between CMC and CMC-PA electrodes becomes very obvious. While both electrodes show a stable cycling performance, higher discharge capacities are delivered from the CMC-PA electrode. At high C-rates (2C, 3C and 5C), the difference is even more pronounced. At 5C (Figure 5a; 30th cycle), 144 mAh·g^−1^ and 124·mAh g^−1^ were delivered by the CMC-PA and CMC electrodes, respectively. The lower discharge capacities of the CMC-based electrode are mostly related to the lower conductivity of the loosely-packed coated layer.

Upon the addition of PA in the slurry (Figure 5a), the electrodes exhibit remarkable electrochemical performance, even at high C-rates. Additionally, the cells showed excellent capacity retention after five cycles at 5C and the subsequent cycles at 1C. However, the LTO-CMC-PA electrode shows a lower rate capability compared to those based on GG-PA and pectin-PA. This could be related to the higher electrolyte uptake of, e.g., guar gum compared to CMC [9,10]. In fact, the motion of the galactomannan and the ether oxygens’ lone-pair electrons of the GG molecule coordinate Li^+^ ions in its structure, comparable to polyethylene oxide (PEO) in solid electrolytes, which accounts for the improvement in electrochemical performance [17,18]. However, CMC-PA electrodes show the highest value of adhesion strength, due to the optimal binding of the solid material components resulting from the homogeneous binder distribution, i.e., the optimal electrode preparation. Thus, it is reasonable to assume that the better electrochemical performance of LTO electrodes made with guar gum and pectin is rather related to increased ionic conductivities than solid particle adhesion. Additionally, pectin-PA and GG-PA electrodes show comparable electrochemical performance at low C-rates of 0.1C, 0.5C and 1C. However, at 2C, 3C and especially 5C, electrodes using pectin as the binder showed the highest capacity. As the adhesion strength is obviously not directly correlated with the electrochemical properties of the herein presented electrodes, the best performance of pectin electrodes has to be related to its different molecular structure and its affinity towards the electrolyte favoring lithium ion transport.

Figure 5b displays the discharge capacity and the coulombic efficiency of a cathode-limited, lithium-ion cell consisting of NMC and LTO electrodes, both made using CMC as the binder and PA as the pH-modifier. Besides the first cycle at low current density (0.1C), the test was performed at constant charge/discharge current densities of 1C. The cells showed an average discharge capacity of ~120 mAh·g^−1^ in the course of 190 consecutive charge/discharge cycles at a 1C rate. Moreover, high values of coulombic efficiency (~99.8%) were achieved. These results confirm the validity of making NMC and LTO electrodes using CMC and PA as the binder and the pH-modifier, respectively, resulting in fully-aqueous processed LIBs with remarkable performance.

## 4. Conclusions

The reported results prove the applicability of polymers from renewable sources as binders for LTO electrodes, using PA as the pH-modifier. A small addition of the latter, in fact, leads to a great performance improvement due to optimal composite electrode cohesion and adhesion to the current collector.

In addition to carboxymethylcellulose, two natural binders, guar gum and pectin, were evaluated for making LTO electrodes. Regarding the thermal stability, the decomposition of pectin was detected near 200 °C, while GG and CMC are stable up to 250 °C. Thus, all three binders can support high temperature drying (180 °C). Electrodes prepared using GG showed the lowest adhesion strength, mostly due to its branched mannose polymer chain. CMC-PA electrodes (linear cellulose chain) were more adhesive than pectin (linear galacturonic acid chain). The effect of the polymeric chain was also evident in the electrochemical test: guar gum- and pectin-based electrodes showed a slightly superior rate capability compared to CMC electrodes. As discussed, the GG molecule offers a better affinity for the organic electrolyte than CMC. Additionally, the binder ability to coordinate Li^+^ affects the overall electrochemical performance. The Li^+^ coordination by GG and pectin polysaccharides may be higher than that of the CMC molecule due to the lower motion of the linear cellulose chain. Moreover, the pectin polymer might also have good affinity to organic electrolytes, such as the GG molecule, due to the α-linking between the galacturonic acid rings, resulting in higher electrolyte absorption.

Finally, full lithium-ion cells were manufactured using NMC and LTO electrodes both prepared via the aqueous process using CMC as the binder and PA as the pH-modifier. The full-cell delivered a stable and remarkable discharge performance of ~120 mAh·g^−1^ at 1C over 190 cycles with high coulombic efficiency (99.8%). These results reaffirm the suitability of making LIB electrodes by simple and inexpensive aqueous processes.

## Supplementary Materials

Supplementary Materials can be found at www.mdpi.com/2073-4360/8/8/276/s1.
