# Supplementary material for: Study of Water-Based Lithium Titanate Electrode Processing: The Role of pH and Binder Molecular Structure
Source: Polymers (Basel). 2016 Aug 2;8(8):276. doi: 10.3390/polym8080276 (PMC6431947; doi:10.3390/polym8080276)
Supplement: Supplementary file 1 [file polymers-08-00276-s001.pdf]

Supplementary Materials: Study of Water-Based Lithium Titanate Electrode Processing: The Role of pH and Binder Molecular Structure

Diogo Vieira Carvalho, Nicholas Loeffler, Guk-Tae Kim, Mario Marinaro, Margret Wohlfahrt-Mehrens and Stefano Passerini

The pH measurements were carried out with a Lab 860 pH meter (SI Analytics) using a Blue Line 18 pH electrode (Schott Instruments) at a temperature of 24 ± 2 °C. The experiments were conducted using only the active material LTO in water and phosphoric acid (PA, Bernd Kraft GmbH) since the final slurry pH is not influenced by the conductive carbon and binder. The pH measurements started with only deionised water and subsequently the LTO powder (40 wt % of water) was added. A second experiment was done using PA (1 wt % of LTO) prior the LTO addition. The slurries were stirred for 150 min while the pH evolution was detected.

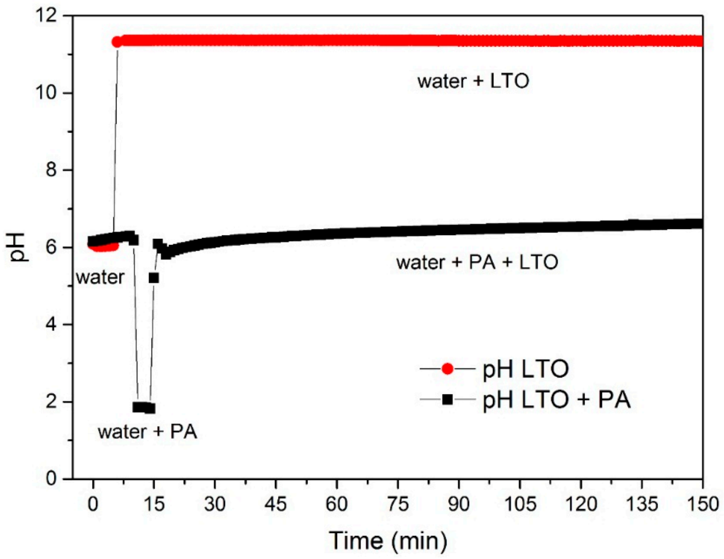

Figure S1. pH evolution of the aqueous slurry containing LTO active material (circles) and phosphoric acid (squares).

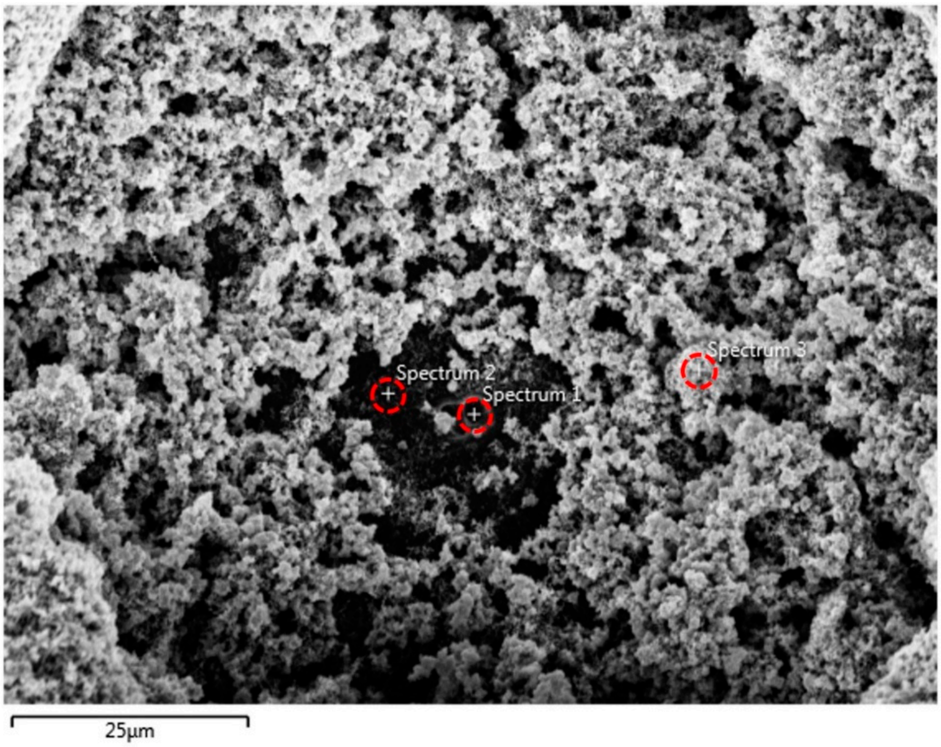

Figure S2. SEM image of the electrode prepared using CMC as binder without PA (LTO-CMC) showing three spots where EDX experiments were performed.

Table S1. Element fractions estimated by EDX measurements on electrode surface prepared without PA (LTO-CMC).

Spectrum 1	Atomic %	Spectrum 2	Atomic %	Spectrum 3	Atomic %
C	8.80	C	28.19	C	84.09
O	3.50	O	5.31	O	10.09
Al	87.69	Al	66.12	Al	1.20
Na	0	Na	0	Na	0.45
Ti	0	Ti	0.38	Ti	4.16
Total	99.99	Total	100	Total	99.99

© 2016 by the authors; licensee MDPI, Basel, Switzerland. This article is an open access article distributed under the terms and conditions of the Creative Commons Attribution (CC-BY) license (http://creativecommons.org/licenses/by/4.0/).
